# Supplementary material for: LRRK2 interactions with microtubules are independent of LRRK2-mediated Rab phosphorylation
Source: EMBO Rep. 2025 May 27;26(13):3445–66. doi: 10.1038/s44319-025-00486-6 (PMC12238589; doi:10.1038/s44319-025-00486-6)
Supplement: Supplementary file 1 — Appendix [file 44319_2025_486_MOESM1_ESM.pdf]

LRRK2 interactions with microtubules are independent of LRRK2-mediated Rab phosphorylation

Tuyana Malankhanova, Zhiyong Liu, Enquan Xu, Nicole Bryant, Ki Woon Sung, Huizhong Li, Samuel Strader, Andrew B. West*

Duke Center for Neurodegeneration Research, Department of Pharmacology and Cancer Biology, Duke University, Durham, NC, USA

Appendix

Table of contents

Appendix Figure S1 Pg. 2

Appendix Figure S2 Pg. 4

Appendix Figure S3 Pg. 5

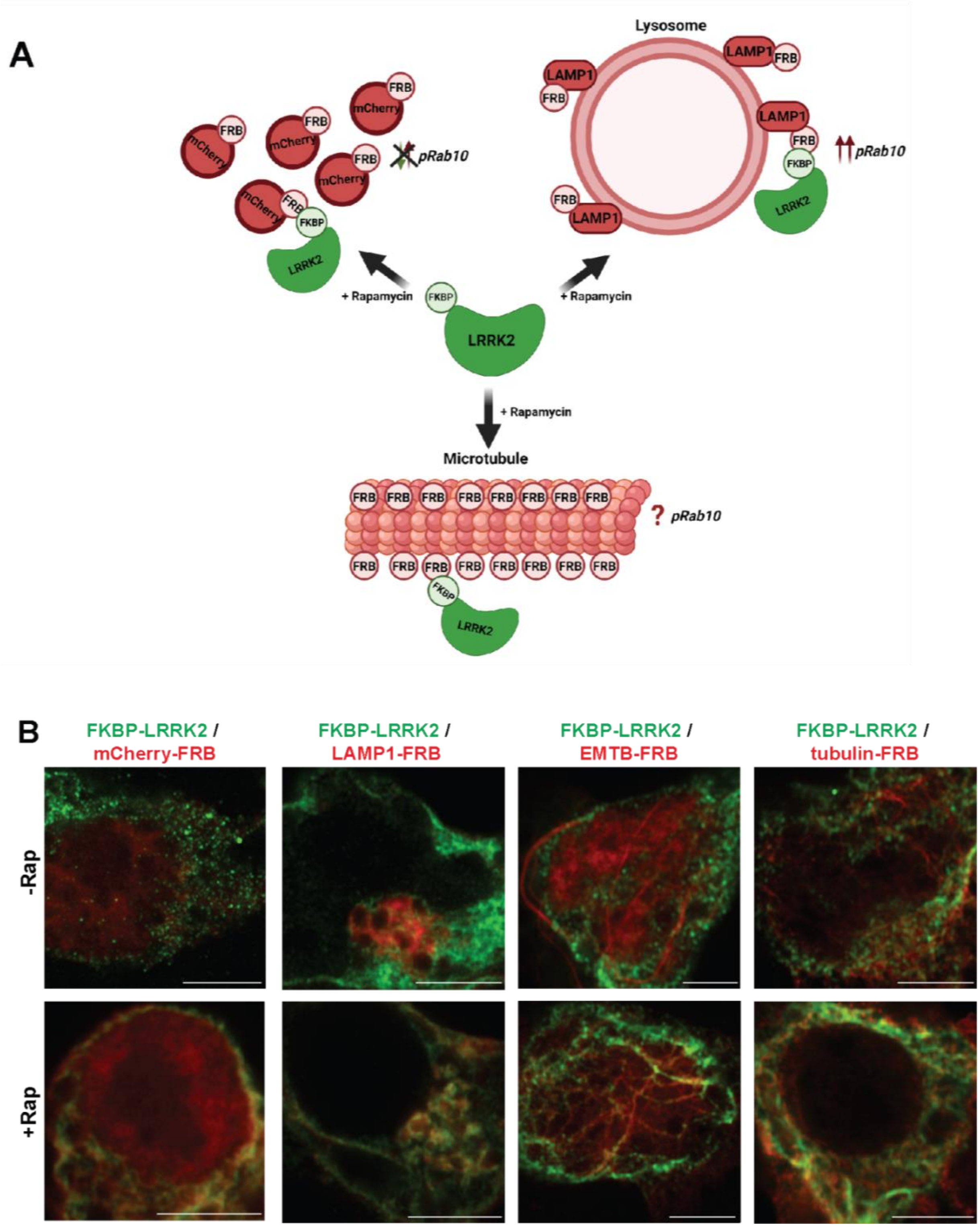

Appendix Figure S1. FKBP-LRRK2 is trapped on microtubules with EMTB-FRB or tubulin-FRB with rapamycin treatment.

A. Schematic representation of FKBP-fused full-length LRRK2 protein trapping to FRB-fused proteins localized diffusely in the cytosol (mCherry-FRB), at lysosomes (LAMP1-FRB), along

microtubules (EMTB-FRB) or with tubulin, both free ß-tubulin and polymerized (tubulin-FRB). The trapping is rapidly induced by rapamycin supplementation.

B. Representative confocal images of cells co-transfected with FKBP-WT-LRRK2 (green) and the indicated FRB-fused protein (red), with or without rapamycin (100 nM for 30 min). Magnified views shown here correspond to selected regions of the same cells shown in lower magnification in Figure 3A-D. Scale bars: 10 µm.

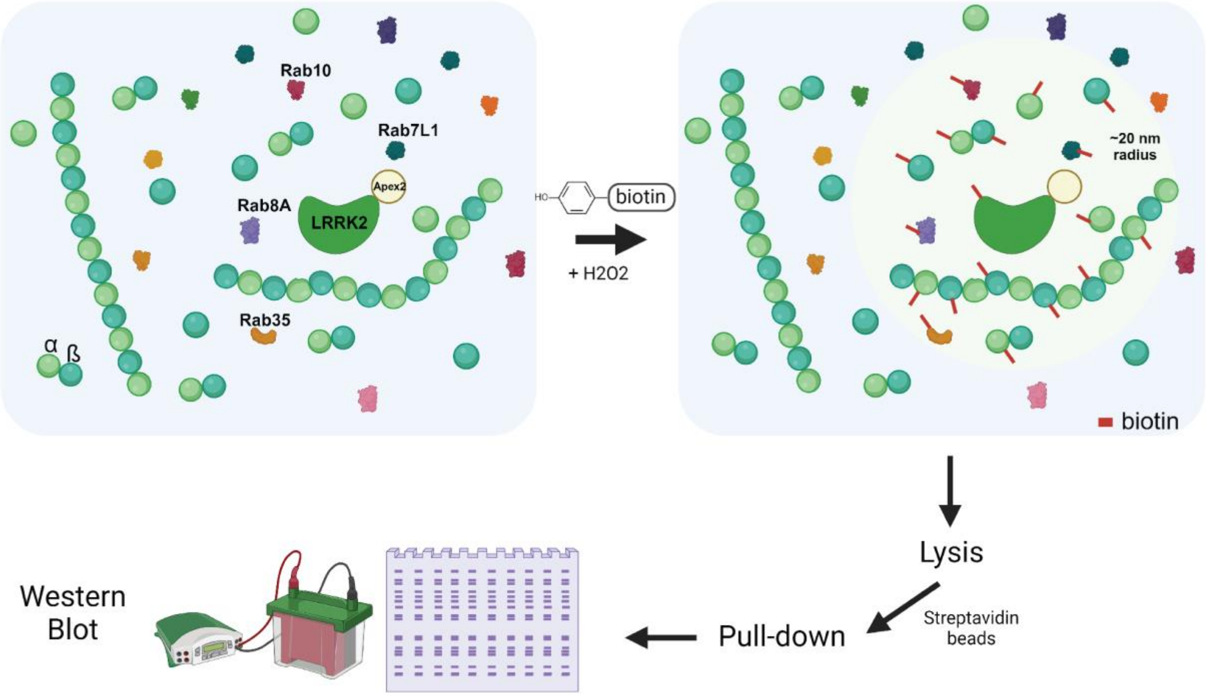

Appendix Figure S2. Biotinylation workflow for the quantitative analysis of proteins in proximity to FLAG-APEX2-LRRK2.
Schematic representation of APEX2-mediated biotinylation of LRRK2-proximity and interacting proteins. APEX2 rapidly catalyzes biotin-phenol oxidation with H_2O_2 resulting in proximity protein labeling with biotin. It is expected that biotinylated proteins are up to 20 nm away from the APEX2 fused protein in the cell. After cell lysis, biotinylated proteins are purified using streptavidin beads and analyzed by western blots or label-free whole proteomics with LC/MS/MS. As an informative control, the cytosolic FLAG-APEX2 protein (without LRRK2) is analyzed in parallel.

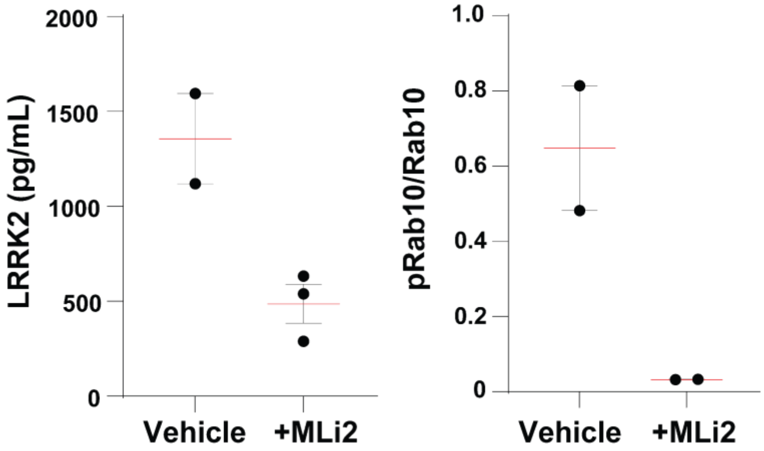

Appendix Figure S3. Confirmation of successful LRRK2 inhibition with oral-gavage treatment of mice with the MLi2 LRRK2 inhibitor.

Using protocols and approaches previously described (Yuan et al., Mol Neurodegener. 2024 Jun 11;19(1)) for the measurements of serum LRRK2 and pRab10 to total Rab10 ratios after MLi2 oral gavage, here, serum from mice used for immunohistochemical detection of LRRK2 (main Fig. 6C, D) was procured and measured for LRRK2 (pg per mL of serum) and the ratio of pT73-Rab10 to total Rab10 (in serum). After MLi2 treatment, the ratio of pRab10 to total Rab10 levels dropped to undetectable levels 2 h after oral dosing of MLi2 (immediately before perfusion and preparation for immunohistochemistry), and LRRK2 levels diminished more than 50%, consistent with past observations with this dosing regimen in mice.
