# Supplementary material for: LRRK2 interactions with microtubules are independent of LRRK2-mediated Rab phosphorylation
Source: EMBO Rep. 2025 May 27;26(13):3445–66. doi: 10.1038/s44319-025-00486-6 (PMC12238589; doi:10.1038/s44319-025-00486-6)
Supplement: Supplementary file 3 — Expanded View Figures [file 44319_2025_486_MOESM3_ESM.pdf]

Expanded View Figures

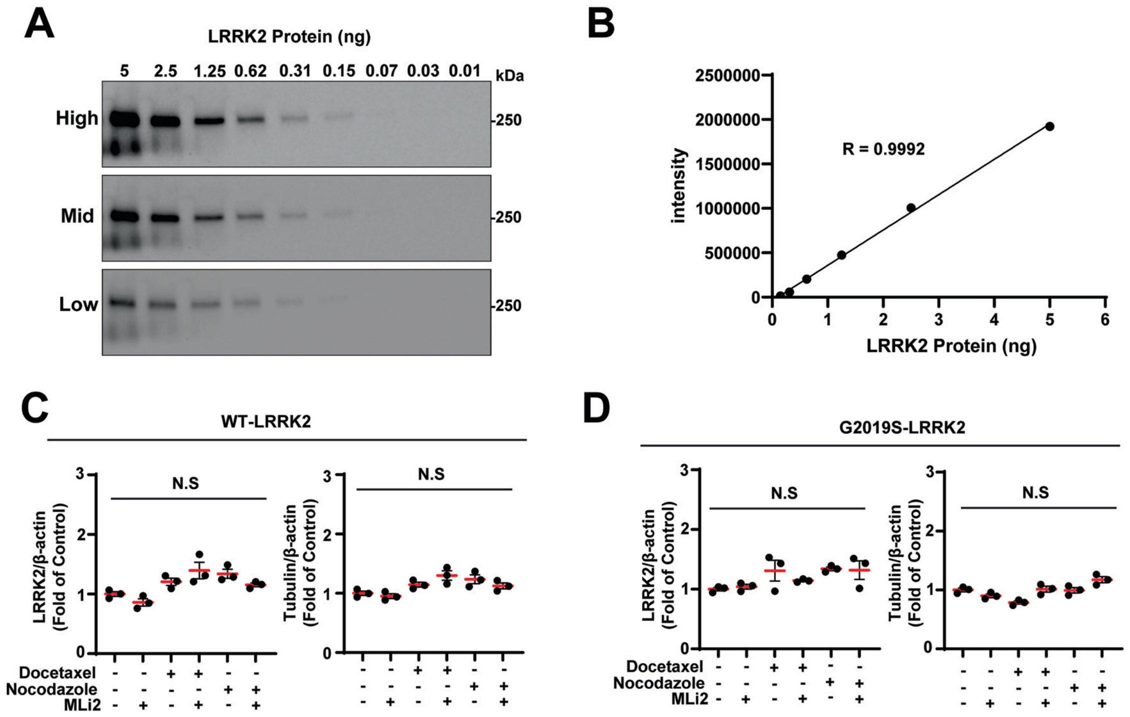

Figure EV1. LRRK2 levels in macrophages do not change with docetaxel, nocodazole, or MLi2 treatments.

(**A**, **B**) Representative immunoblots of LRRK2 protein diluted two-fold to lower limits of detection, with a scatter plot highlighting good linearity (r = 0.99) of measured band intensities across the range of signals produced in the immunoblotting approaches used in this study. (**C**, **D**) Quantification of the relative (fold of non-treated cells) ratio of LRRK2 to ß-actin signals from immunoblots in Fig. 1A-B, where each dot represents immunoblot analysis of one biological replicate. Cells were treated with docetaxel (10 µM), nocodazole (10 µM), and MLi2 (250 nM) prior to lysis. Red bars in the column graphs show mean with ± SEM error bars. Statistical significance was assessed by one-way ANOVA; N.S, not significant.

 © The Author(s)

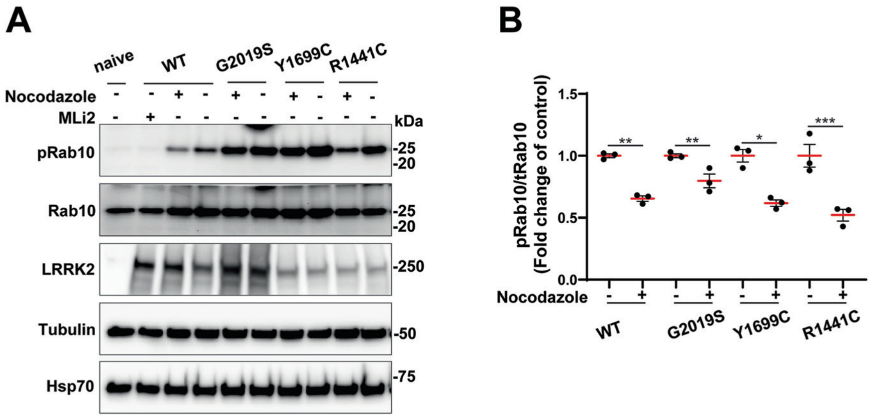

Figure EV2. Nocodazole treatment partially inhibits transfected LRRK2 phosphorylation of endogenous Rab10 in HEK293T cells.

(**A**, **B**) Representative immunoblots of HEK293T cell lysates transfected with the indicated LRRK2-expressing plasmid, treated with or without nocodazole (10 µM) and MLi2 (250 nM) for 2 h prior to lysis. Relative (fold of vehicle control) transfected cells for the quantification of the ratio of pRab10 to total Rab10, where each dot represents immunoblot analysis of one biological replicate ($n = 3$ biologically independent experiments). Group means are shown. Error bars represent ± SEM. Significance was assessed by Student's *t*-test with * representing $P < 0.05$, ** representing $P < 0.01$, *** representing $P < 0.001$. Exact *P* values from left to right: $P = 0.0093$; $P = 0.0025$; $P = 0.0242$; $P = 0.0002$.

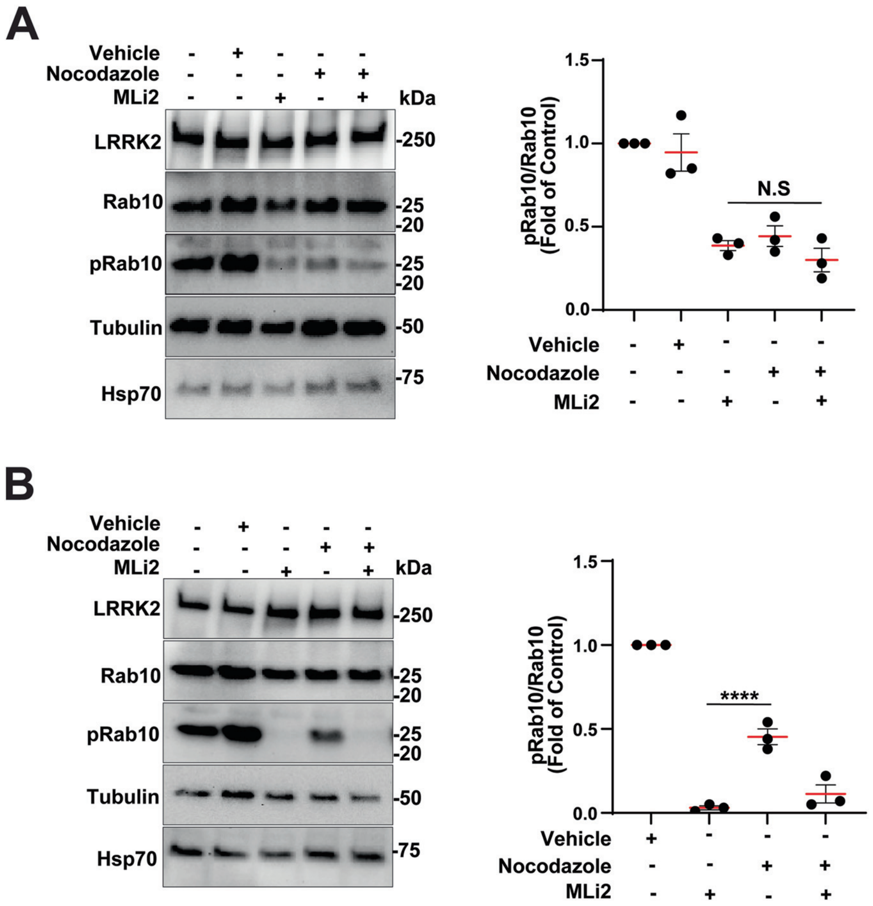

Figure EV3. Nocodazole treatment does not hasten reductions of pRab10 after LRRK2 kinase inhibition.

(**A**) Representative immunoblots of WT-LRRK2 BMDM cell lysates. Cells were treated with nocodazole (10 μM) and MLi2 (250 nM) for 2 min prior to lysis. Relative (fold of non-treated cells) quantification of the ratio of pRab10 to total Rab10 is shown, where each dot represents immunoblot analysis of one biological replicate ($n = 3$ biologically independent experiments). Group means are shown. Error bars represent ± SEM. Significance was assessed by one-way ANOVA with Tukey's *post hoc* test; N.S, not significant. (**B**) The same experiment as described in (**A**), but with a 5 min exposure of drugs (nocodazole and/or MLi2 as indicated), there is a partial loss of pRab10 caused by nocodazole treatment. Each dot represents one biological replicate ($n = 3$ biologically independent experiments). Group means are shown. Error bars represent ± SEM. Significance was assessed by one-way ANOVA with Tukey's *post hoc* test with **** representing $P < 0.0001$.

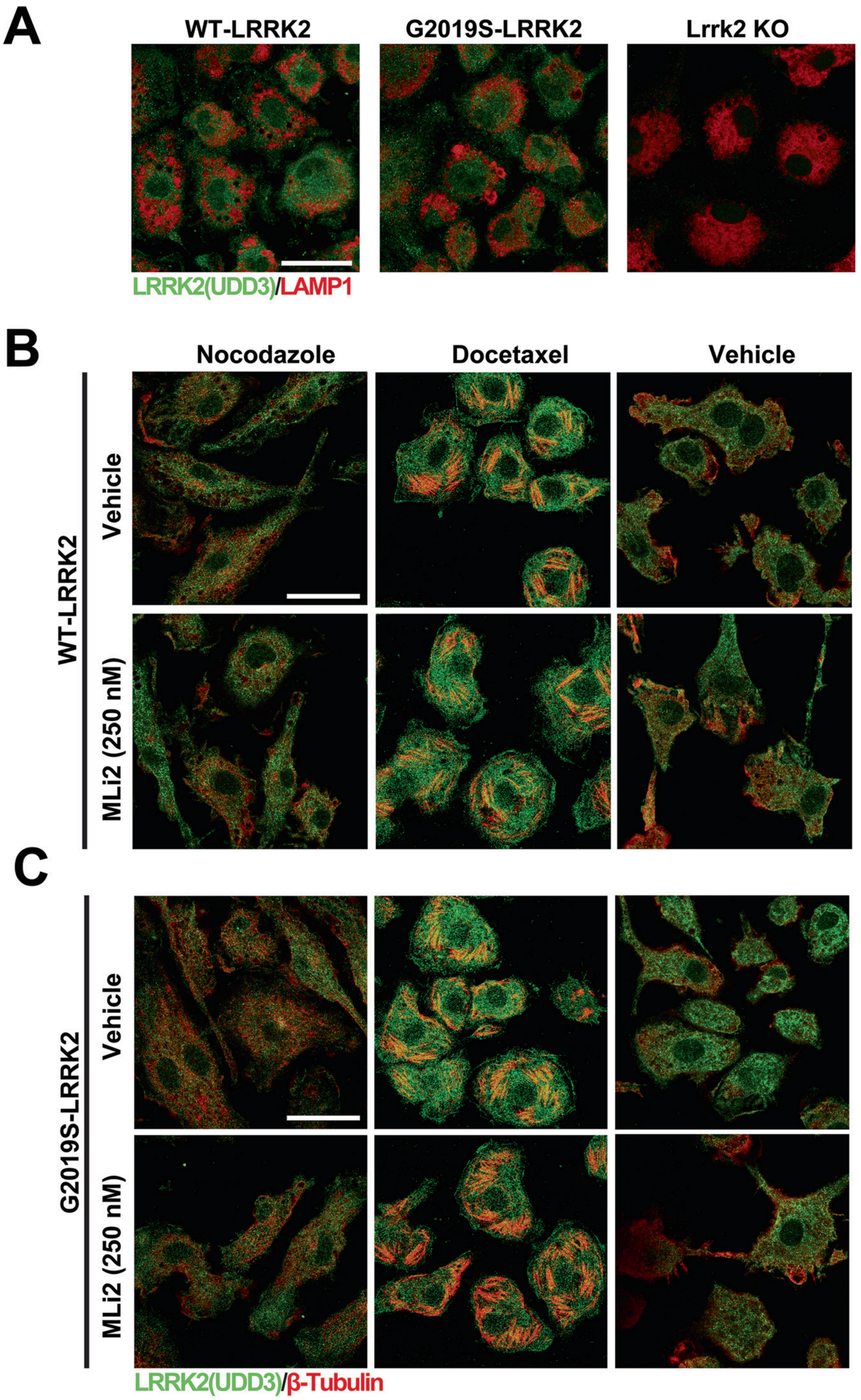

Figure EV4. Nocodazole or docetaxel treatment does not change endogenous LRRK2 protein distribution in macrophages.

(**A**) Representative immunocytochemistry of LRRK2 stained with the N-terminal targeting UDD3 antibody (green) and LAMP1 (red) from BMDM procured from WT-LRRK2, G2019S-LRRK2, or *Lrrk2* knockout mice. Cells were treated with docetaxel or nocodazole (10 μM, 2 h), then paraformaldehyde-fixed and saponin-treated prior to staining. There was no detectable LRRK2 signal from UDD3 in *Lrrk2* knockout macrophages, indicating specificity of the antibody in this immunocytochemistry protocol. Scale bars indicate 10 μm. (**B**, **C**) Representative immunocytochemistry showing endogenous WT-LRRK2 or G2019S-LRRK2 distribution is not affected by MLi2 (250 nM for 2 h) with or without docetaxel (10 μM) or nocodazole (10 μM), also treated for 2 h. No instances of LRRK2-skein-like structures or large (e.g., >1 μM) aggregates were noted in any cell in the experiments. Scale bars indicate 10 μm.

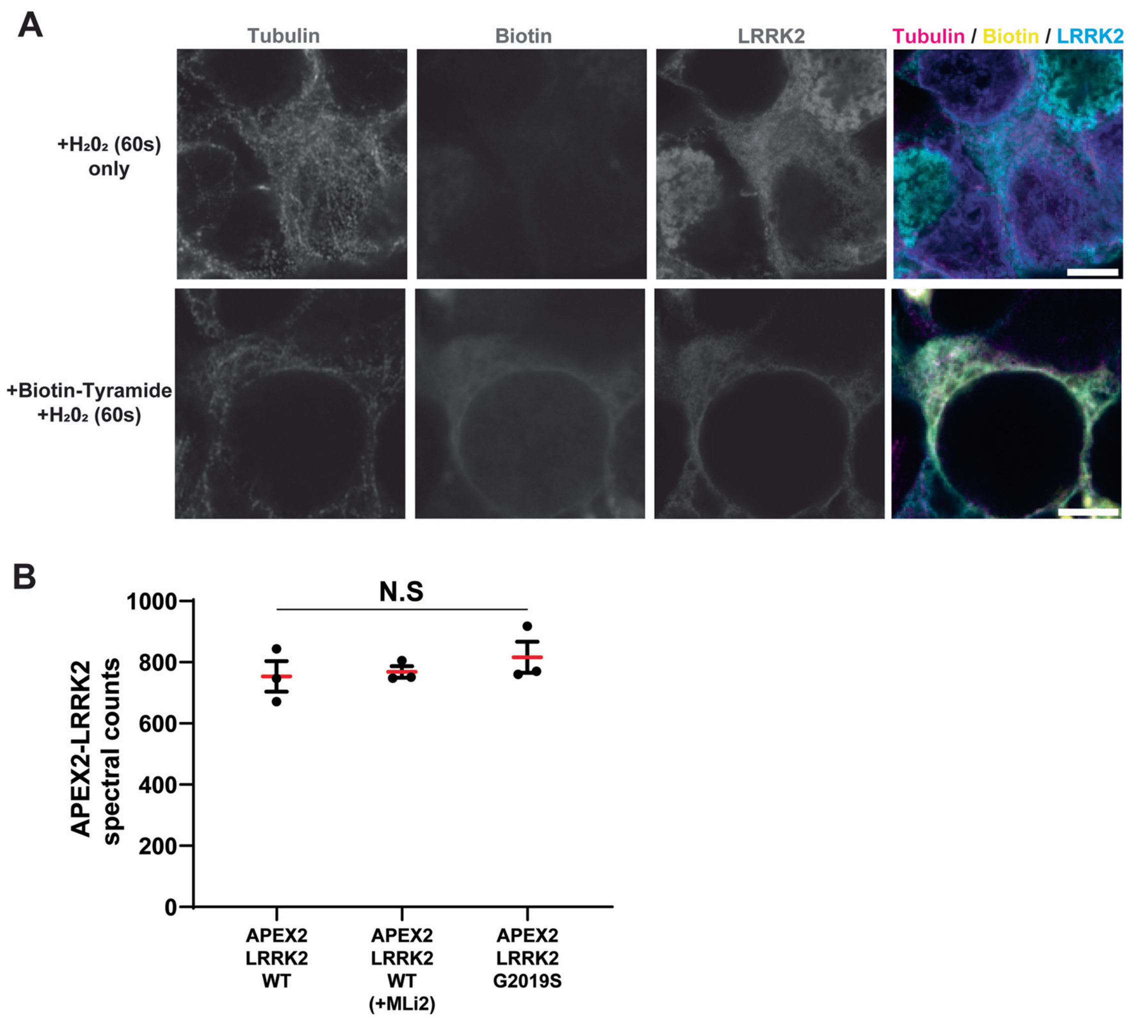

Figure EV5. Subcellular localization and efficiency of APEX2-LRRK2 proximity labeled proteins.

(**A**) Representative immunocytochemistry of transfected HEK293T cells with FLAG-APEX2-WT-LRRK2 plasmid, with and without biotin-tyramide and H_2O_2 supplementation. H_2O_2 treatment on its own did not result in measurable biotinylated proteins, whereas biotinylated proteins were measured when biotin-tyramide was included. Scale bars: 10 µm. (**B**) The overall concentration of biotinylated proteins captured with pull-downs from 3 biological replicates did not vary according to the presence of the G2019S-LRRK2 mutation or the LRRK2 inhibitor MLi2. Red bars in the column graphs show group means with ± SEM error bars. Statistical significance was assessed by one-way ANOVA; N.S, not significant.

 © The Author(s)
